# Predicting fitness coaches’ intentions to include persons with disabilities in gyms: an application of the theory of planned behavior

**DOI:** 10.3389/fspor.2025.1608703

**Published:** 2025-07-24

**Authors:** Milan Dransmann, Michael Braksiek, Christopher Meier, Bernd Gröben, Lara Lesch, Pamela Wicker

**Affiliations:** ^1^ Department of Sports Science, Bielefeld University, Bielefeld, Germany; ^2^ Department of Sports Science, University of Vechta, Vechta, Germany; ^3^ Central Operating Unit Sport and Exercise (ZB-SB), University of Siegen, Siegen, Germany

**Keywords:** attitude, subjective norm, perceived behavior control, intention, confirmatory factor analysis—CFA, structural equation model, regression analysis

## Abstract

**Introduction:**

Promoting equal access to fitness offers implies the inclusion of individuals with disabilities in gyms. However, many gyms do not meet the needs of people with disabilities due to physical, social, and human resource barriers. This study examines fitness coaches’ capacities and intentions of providing fitness offers for individuals with disabilities using the theory of planned behavior as a framework.

**Methods:**

A quantitative online survey was conducted with 182 fitness coaches in Germany. The data were analyzed using confirmatory factor analysis, structural equation modeling, and regression analysis to empirically test the theory of planned behavior constructs and identify factors influencing coaches’ intentions.

**Results:**

The analysis confirmed that descriptive norms and experiential attitudes predict coaches’ intentions to support individuals with disabilities, highlighting the importance of social pressures and experience-based attitudes. Previous experiences, frequent contact with individuals with disabilities, and adequate preparation, often achieved through informal means, play crucial roles in shaping these intention-forming factors.

**Discussion:**

Although fitness coaches express strong intentions to support individuals with disabilities, there is a need for targeted training and resources. These trainings and additional resources could equip coaches with the necessary skills and knowledge to effectively translate their intentions into practice, even though the actual implementation was not measured in the study.

## 1 Introduction

The United Nations Convention on the Rights of Persons with Disabilities (CRPD) explicitly includes the right to participate in “mainstream sporting activities” (1). This international mandate underscores the importance of creating inclusive environments where individuals with disabilities can participate in physical activities. In this context, gyms play a crucial role as they are widely available venues that offer opportunities for exercise and social interaction. Exercising in a gym can benefit one's mental health, well-being, and social affiliation (2). In Germany, exercising in a gym qualifies as a mainstream sporting activity, with gym memberships reaching 10.3 million in 2022, meaning that 12.3 percent of the total population have a gym membership (3). However, most gyms currently do not meet the requirements of people with disabilities. Sharon-David et al. (4) identify accessibility, oppressive attitudes, and a lack of social support provided by family members, friends, healthcare professionals, and gym staff (e.g., coaches) as main barriers.

Kennedy et al. (5) highlight person-based support as the most fundamental form of social support for successfully integrating individuals with disabilities into gyms. The authors state that gym staff and coaches are responsible for delivering this support, typically in one-on-one or small group settings, which differs from common gym practice. While coaches are compensated for their work, their positive and effective support depends on their intention to assist people with disabilities. Since attitudes, subjective norms, and perceived behavioral control comprise behavioral intention (6), it is crucial to consider these factors when analyzing coaches’ intentions.

Existing studies have mostly examined the perspectives of individuals with disabilities or the organizational viewpoint, with limited focus on the role of coaches. Furthermore, the studies that do exist are often qualitative in nature. This highlights a gap in the literature concerning the quantitative analysis of coaches' intentions in this context. By addressing this gap, the study contributes to a better understanding of how to foster environments that enhance the positive impact of person-based support in gym settings.

This study investigates the inclusion of people with disabilities in gyms from the perspective of coaches. It addresses two research questions: (1) which factors influence the intention of fitness coaches to supervise people with disabilities in the gym? and (2) which contextual factors predict these intention-forming factors? The study examines these questions through a quantitative analysis of an online survey completed by gym coaches. By applying the theory of planned behavior (6), which is particularly suitable for analyzing behavioral intentions and their antecedents, such as attitudes, social norms, and perceived control (7), the study contributes to the literature by exploring gym coaches' perspectives on the inclusion of people with disabilities in the gym environment. This approach provides a robust framework for understanding the external and social influences on coaches' behavioral intentions, complementing the internal focus of other theories such as self-determination theory (8).

## 2 Theoretical framework and literature review

In this chapter, the theoretical framework (i.e., the theory of planned behavior), the current state of research on the topic of inclusion of people with disabilities in the gym environment, and the hypotheses are presented. The state of research is systematized along three levels: the individual level (focused on people with disabilities), the organizational level (focused on the organizational perspective), and the intermediary level (focused on coaches in gyms).

### 2.1 Theory of planned behavior

Fishbein and Ajzen (6) formulated the theory of planned behavior (TPB) as a foundational framework to predict and understand human behavior across various domains. The TPB evolved from the earlier theory of reasoned action (9) and incorporates perceived behavioral control to address behaviors not entirely under volitional control. This framework extensively applies to fields such as health, education, and environmental practices (10).

The TPB posits that an individual's behavior primarily depends on the intention to perform that behavior. Three core components influence this intention: attitude toward the behavior, subjective norms, and perceived behavioral control. Attitude reflects how favorably or unfavorably a person evaluates the behavior (6). It is widely acknowledged that attitudes toward a behavior encompass both instrumental aspects, such as being seen as desirable or undesirable and valuable or worthless, and experiential aspects, such as being perceived as pleasant or unpleasant and interesting or boring (7). According to Fishbein and Ajzen (6), measures of attitudes should contain items representing these two sub-components.

Subjective norms involve pressures from others to perform or not perform the behavior (7). These norms may include expectations from supervisors, colleagues, friends, family, and society. Similar to attitude, there is a differentiation between two sub-components: injunctive norms, which are perceptions of what others think one should do, and descriptive norms, which are perceptions of what others are doing (11). To fully capture the essence of subjective norms, Fishbein and Ajzen (6) emphasize the importance of designing measurement items that address both types of norms. Understanding both types of norms is crucial, as they can either facilitate or hinder inclusive practices (12) in the gym environment.

Perceived behavioral control, similar to Bandura's (13) concept of self-efficacy, refers to the perceived ease or difficulty of performing a certain behavior, shaped by past experiences and anticipated obstacles. According to Fishbein and Ajzen (6), there are two identifiable factors for perceived behavioral control. Items focusing on how easy or difficult a behavior is to perform, or on one's confidence in the ability to perform it, generally align with one factor. Conversely, items addressing the degree of control over the behavior, or how much the behavior's execution depends on the individual, generally align with another factor. The exact nature of the two empirically identified factors remains unclear. The review of Ajzen (14) suggests that items representing these factors are correlated, and, when measures incorporate both types of items, they consistently demonstrate high internal consistency. Thus, similar to the measurement of attitudes and subjective norms, a comprehensive measure of perceived control is achieved by including items that represent both factors in empirical studies (6). These factors are usually labeled as capacity and autonomy: Capacity reflects an individual's perception of the ease or difficulty of conducting a behavior, which is noted to be overlapping with self-efficacy (15). Autonomy refers to the extent to which an individual believes the performance of the behavior is up to them (16).

Overall, the TPB suggests that favorable attitudes and subjective norms, along with high perceived behavioral control, strengthen intentions to perform the behavior (10). Based on evidence of discriminant validity (17), an increasing number of studies have tested the six lower-level constructs (instrumental attitude, experiential attitude, injunctive norm, descriptive norm, capacity, autonomy) as predictors of both intention and behavior (18).

The TPB is a widely applied model across various fields. It has been employed in domains such as health, environment, traffic safety, occupational psychology, social participation, and sports. Cunningham and Kwon (19) explored the application of the theory of planned behavior to understand consumer intentions to attend a sport event, demonstrating that attitudes, subjective norms, and perceived behavioral control, when combined with previous behavior, significantly predict these intentions. Kim and James (20) demonstrated that the theory of planned behavior effectively predicts intentions to purchase sport team licensed merchandise, with attitudes, subjective norms, and perceived behavioral control accounting for 64% of the variance, and additional factors like past behavior and role identity enhancing predictive power. Liao et al. (21) integrated the theory of planned behavior and self-determination theory to predict sports participation and exercise intentions among college students in China, finding that attitudes and perceived behavioral control significantly impact intentions, while satisfying psychological needs indirectly influences participation through various mediators. The findings underscore the theory's effectiveness in the sports context, providing insights for both theoretical extensions and practical applications.

Focusing on the inclusion of individuals, TPB has provided valuable insights into promoting inclusive behaviors, particularly in educational environments (22). Attitudes are a crucial determinant of inclusive practices. Studies involving physical education teachers, such as those by Tripp and Rizzo (23), indicate that positive attitudes toward students with disabilities lead to more inclusive educational practices. This study emphasizes the importance of fostering favorable attitudes through targeted education and exposure to the benefits of inclusive practices. Additionally, analysis suggests that longer professional experience as a teacher correlates with more positive attitudes toward inclusion, aligning with findings from Oh et al. (24) and Tiwari et al. (22).

Social norms were found to play a central role in the adoption of inclusive measures. Research in educational settings demonstrates that when a supportive community and school leadership emphasize inclusivity, educators are more inclined to adopt such practices (25, 26). This highlights the critical role of a community that values inclusivity at all levels.

Perceived behavioral control is essential for translating intentions into actual inclusive behavior. Jeong and Block (27) conducted research with physical education teachers, revealing that providing sufficient resources and training is vital for overcoming barriers to inclusion in school settings. Educators who feel capable and equipped to adapt their teaching methods are more likely to implement inclusive practices. Their study further suggests that higher qualifications, alongside extensive experience, enhance perceived behavioral control by boosting confidence and capacity to implement such practices.

In the context of coaching, Sagas et al. (28) applied TPB to predict head coaching intentions of male and female assistant coaches, revealing that attitudes, subjective norms, and perceived behavioral control are significant predictors. The study found notable gender differences, with female coaches scoring lower than male coaches on intentions, attitudes, and subjective norms, highlighting the potential for targeted interventions to address these disparities. Rigby et al. (29) found that the theory of planned behavior effectively predicts athletic coaches' compliance with concussion-management guidelines, suggesting its potential applicability to coaches. The results showed that attitudes and perceived behavioral control significantly influenced behavioral intentions, while perceived behavioral control and behavioral intentions predicted actual compliance behavior, whereas subjective norms did not. Chrisman et al. (30) used the theory of planned behavior to explore how coaches in youth football and soccer communicate about contact, highlighting that while coaches recognize their responsibility to guide athletes in reducing injury risk, they face barriers such as insufficient institutional guidance and concerns about creating fear. The findings emphasize that attitudes, norms, and perceived behavioral control influence coach communication, suggesting the need for programs that support positive communication strategies as a means of concussion prevention.

In the fitness domain, Kasser and Rizzo (31) examined the intentions of fitness coaches to provide exercise opportunities for individuals with multiple sclerosis. This study, conducted in gym settings, found that coaches' attitudes and perceived competence are significantly associated with their intentions to develop programs that are both inclusive and health-promoting. However, the study had several limitations, such as a narrow focus on a specific condition (multiple sclerosis). This limitation highlights the need to explore a broader perspective, examining how fitness coaches view the inclusion of individuals with disabilities in gyms more generally, i.e., beyond specific conditions.

### 2.2 Inclusion of people with disabilities in gyms

Using the theory of planned behavior (9) as a theoretical framework for understanding behavioral intentions makes it essential to explore current research on the inclusion of people with disabilities in gym environments. This chapter explores the current research across three distinct levels: the individual level, the organizational level, and the intermediary level, particularly focusing on the role of fitness coaches.

At the individual level, people with disabilities face significant barriers that affect their participation in gym-based physical activities. Physical accessibility remains a primary concern, as many gyms lack appropriate equipment and facilities to accommodate diverse needs of people with disabilities. Sharon-David et al. (4) identified non-accessible entrances and inadequate equipment as major constraints. These physical barriers often compound with a lack of knowledge about how to modify exercises or use equipment safely and effectively (32). Beyond structural barriers, psychological factors also play a crucial role. People with disabilities report that they feel unwelcome or marginalized in gym environments, leading to decreased self-esteem and motivation to engage in physical activities. However, increased contact with individuals with disabilities can foster greater understanding and empathy, helping to reduce these psychological barriers (33). Social support plays a crucial role in overcoming these challenges. Kennedy et al. (5) emphasized that personalized support significantly enhances the self-efficacy of individuals with disabilities, making them feel more integrated into the fitness community. This personalized support, through frequent contact with individuals with disabilities, can indirectly influence injunctive and descriptive norms, thereby fostering a more socially accepted environment for inclusive practices. Exercising alongside others and receiving their practical help was linked to increased engagement in physical activities (34). The presence of someone with whom to connect in the gym setting (i.e., validation support) was highlighted in three studies as a key factor that encourages participation in gym-based exercise (35–37).

At the organizational level, gyms should address both physical infrastructure and social dynamics to create inclusive environments. The study by Lesch et al. (38) highlights that gym managers perceive the inclusion of people with disabilities as an important societal issue but do not consider people with disabilities a relevant target group due to perceived barriers such as the size of the target group, lack of demand, lack of profitability, and potential (negative) impact on the gym's image. These perceptions, as well as several capacity issues, seem to hinder the development of inclusive practices in German gyms. Training and education of gym staff play a crucial role in fostering an inclusive culture within gyms. Rimmer et al. (39) underscore the necessity for fitness staff to become well-educated and sensitive to the needs of individuals with disabilities, including understanding disability etiquette, adapting exercises, and recognizing unique challenges. Such enhanced preparation through targeted training programs on the topic of inclusion can significantly shape instrumental and experiential attitudes, emphasizing the importance of tailored educational interventions for fitness staff. Martin Ginis et al. (40) suggest incorporating elements of autonomy, belongingness, and engagement into gym culture, enhancing overall inclusivity.

At the intermediary level, fitness coaches play a pivotal role in shaping inclusive experiences for individuals with disabilities in gym settings. The attitudes and behaviors of coaches significantly influence the level of inclusion achieved. Avramidis and Norwich (41) highlight the importance of positive attitudes in educational settings, which mirrors fitness environments where coaches must adapt programs to meet diverse needs (32). Kasser and Rizzo (31) explored fitness practitioners' intentions regarding exercise programming for individuals with multiple sclerosis, demonstrating the importance of attitudes and perceived competence in promoting inclusive practices. Although this study focused on a specific condition, it underscores the broader need for fitness coaches to receive education in skills and confidence to address the diverse needs of individuals with disabilities and overcome inhibitions or fears. Despite its importance, the intermediary level remains the least explored area in current research. Understanding how fitness coaches can effectively contribute to inclusive gym environments offers significant potential for developing strategies that enhance participation among people with disabilities.

### 2.3 Hypotheses

After explaining the theoretical model and reviewing the state of research, two hypotheses are theoretically developed and empirically tested.

1. Instrumental attitude (H1a), experiential attitude (H1b), injunctive norm (H1c), descriptive norm (H1d), capacity (H1e), and autonomy (H1f) are positively associated with fitness coaches’ intention to supervise individuals with disabilities in gym settings.
2. When the topic of supervision and support for members with disabilities is included in fitness training qualifications (H2a), along with higher levels of qualifications (H2b), enhanced preparation through additional training (H2c), greater experience (H2d), more frequent contact with individuals with disabilities within the gym (H2e), and more frequent contact with individuals with disabilities outside the gym (H2f), attitude is positively influenced.

## 3 Methods

This article is part of a larger research project focused on including people with disabilities in gyms. This article specifically addresses the perspective of coaches by applying the theory of planned behavior to understand their intentions toward fostering inclusive gym environments.

### 3.1 Data collection

Data collection took place from July 2021 to May 2022 using a quantitative online survey. The university's ethics committee approved the questionnaire (registration number: 2021-168-S). The survey targeted individuals with physical or mental disabilities and those with chronic diseases in Germany. Participants had to be at least 16 years old, as this is the minimum age for signing a gym membership contract. The EvaSys software facilitated the survey administration.

The survey link was distributed through two channels. First, emails with the link were sent to German gyms, requesting them to share it with their coaches. Some gyms posted the link on their websites or included it in newsletters. Second, personally known fitness coaches were contacted via email and phone and asked to help distribute the survey link.

Overall, 183 participants completed the questionnaire. However, during the data cleaning process, one case was removed after plausibility checks. The sample consisted of 182 fitness coaches with an average age of 30.78 years, including 89 male and 93 female participants. This balanced gender distribution aligns with general demographic trends in the fitness coaching sector in Germany (42). Regarding the type of gym facility, 26.4% of coaches work in chain-operated facilities with five or more locations, 48.9% are employed in independently operated gyms with one to four locations, 14.3% work in micro-studios with less than 200 square meters and a deliberately limited offer for specific target groups, and 10.4% are based in gyms operated by associations. According to Turulski (43), using data from December 2023 for all of Germany, the distribution of the population across different types of municipalities is as follows: 13.5% live in villages, 26.5% in small towns, 27.6% in medium-sized cities, and 32.4% in large cities. In our study, 12.6% of the coaches work in villages, 25.8% are employed in small towns, 36.8% operate in medium-sized cities, and 24.7% are based in large cities. This suggests that, in terms of their place of residence, the sample of coaches can be considered representative of the general population, with a slight overrepresentation of those in medium-sized cities. Concerning educational background, participants’ qualifications ranged from no formal qualification to a university degree in sports or physiotherapy, with an average educational level of 4.84 on a 6-point scale. This indicates that many participants hold advanced educational qualifications, suggesting that fitness coaches in Germany are formally well-qualified.

### 3.2 Questionnaire and variables

At the beginning of the survey, participants received a detailed introduction explaining the study's purpose, ethical conduct, anonymity of data collection, confidentiality, and data usage for scientific purposes. Participants gave their consent to participate before the survey began. Table 1 provides an overview of all variables used in the analysis. The original questionnaire was administered in German, and an English translation is provided as Supplementary Material to ensure clarity and facilitate future research applications.

**Table 1:** Overview of variables and summary statistics (*n* = 182).

Variable	Description	Mean	SD	Min	Max
*Structure_chain*	Fitness company with five or more facilities (0 = no, 1 = yes)	0.264	—	0	1
*Structure_individual*	Fitness company with one to four facilities (0 = no, 1 = yes)	0.489	—	0	1
*Structure_micro*	Fitness company with less than 200 m^2^ and deliberately limited offer for specific target groups (0 = no, 1 = yes)	0.143	—	0	1
*Structure_association*	Gym of an association (0 = no, 1 = yes)	0.104	—	0	1
*Equipment*	The gym has specific equipment (e.g., training equipment) for people with disabilities (1 = do not agree at all, 7 = fully agree)	2.77	1.72	1	7
*Barrier*	The entrance area (3.8), the training area (3.9), and the sanitary facilities (3.10) of the gym are barrier-free. Mean index of the three items (1 = do not agree at all, 7 = fully agree)	5.33	1.66	1	7
*Qualification*	Qualification of the coach (0 = no formal qualification; 6 = university degree in sports or physiotherapy)	4.84	1.61	0	6
*Topic*	Topic of supervision and support for members with disabilities was covered during fitness training qualification(s) (0 = no, 1 = yes)	0.39	—	0	1
*Preparation*	Other qualifications prepared me for the supervision and support of members with disabilities in the gym (1 = do not agree at all, 7 = fully agree)	2.67	1.53	1	7
*Experience*	Number of years working in a gym	6.32	6.28	0	35
*Contact_gym_frequency*	Frequency of contact with members with disabilities in the gym (1 = less than once a year, 8 = daily)	4.31	2.34	1	8
*Contact_outside_frequency*	Frequency of contact with people with disabilities outside the gym (1 = less than once a year, 8 = daily)	3.76	1.95	1	8
*Gender*	Gender of the respondent (0 = woman, 1 = man)	0.489	—	0	1
*Age*	Age of the respondent (in years)	30.78	11.12	18	69
Attitude	Supervising and supporting members with disabilities in the gym is… (1–7)				
*Attitude_exp_exciting* ^a^	boring—exciting	5.60	1.22	2	7
*Attitude_exp_attractive*	repulsive—attractive	4.95	1.15	2	7
*Attitude_exp_satisfactory* ^a^	unsatisfactory—satisfactory	5.47	1.30	2	7
*Attitude_exp_pleasant*	unpleasant—pleasant	4.92	1.29	2	7
*Attitude_exp_appealing*	disgusting—appealing	5.08	1.08	3	7
*Attitude_exp_harmless* ^a^	frightening—harmless	5.51	1.40	1	7
*Attitude_ins_good* ^a^	bad—good	6.47	1.09	1	7
*Attitude_ins_right*	wrong—right	6.74	0.63	4	7
*Attitude_ins_necessary*	superfluous—necessary	6.55	0.84	2	7
*Attitude_ins_important*	unimportant—important	6.64	0.81	1	7
*Attitude_ins_sensible*	pointless—sensible	6.63	0.79	2	7
*Attitude_ins_favorable* ^a^	unfavorable—favorable	5.96	1.24	2	7
Subjective norm	Please indicate to what extent you agree with the following statements: (1 = do not agree at all, 7 = fully agree)				
*Norm_des_partner*	My partner would supervise and support members with disabilities at the gym.	5.07	1.70	1	7
*Norm_des_supervisor* ^a^	My supervisor would recognize, supervise, and support members with disabilities in the gym.	5.50	1.60	1	7
*Norm_des_family*	My family members would supervise and support members with disabilities at the gym.	5.43	1.40	1	7
*Norm_des_colleagues*	My colleagues would supervise and support members with disabilities in the gym.	5.54	1.29	1	7
*Norm_des_members*	Members of the gym without disabilities would supervise and support members with disabilities in the gym.	4.82	1.42	1	7
*Norm_des_friends*	My friends would include, supervise, and support members with disabilities in the gym.	5.02	1.43	1	7
*Norm_inj_colleagues* ^a^	My colleagues think I should supervise and support members with disabilities in the gym.	4.93	1.65	1	7
*Norm_inj_friends*	My friends think I should supervise and support members with disabilities at the gym.	4.93	1.57	1	7
*Norm_inj_members*	Members of the gym without disabilities think I should supervise and support members with disabilities in the gym.	4.82	1.53	1	7
*Norm_inj_partner*	My partner thinks I should have members, support, and supervise people with disabilities in the gym.	5.16	1.50	1	7
*Norm_inj_supervisor*	My supervisor thinks I should supervise and support members with disabilities in the gym.	5.09	1.55	1	7
*Norm_inj_family*	My family members think I should supervise and support members with disabilities at the gym.	5.30	1.40	1	7
Perceived behavior control (PBC)	Please indicate to what extent you agree with the following statements: (1 = do not agree at all, 7 = fully agree)				
*PBC_aut_time*	I have the time to supervise and support members with disabilities in the gym.	4.47	1.82	1	7
*PBC_aut_responsible*	I am responsible for supervising and supporting members with disabilities in the gym.	4.74	1.82	1	7
*PBC_aut_opportunity*	I have the opportunity to include, supervise, and support members with disabilities in the gym.	4.77	1.82	1	7
*PBC_aut_decision* ^a^	It is my decision to supervise and support members with disabilities in the gym.	4.59	1.76	1	7
*PBC_cap_know*	I know how to supervise and support members with disabilities in the gym.	4.25	1.81	1	7
*PBC_cap_convinced*	I am convinced that I can supervise and support members with disabilities in the gym.	5.07	1.57	1	7
*PBC_cap_skills*	I have the skills to supervise and support members with disabilities in the gym.	4.70	1.65	1	7
*PBC_cap_able*	I am able to supervise and support members with disabilities in the gym.	4.86	1.59	1	7
Intention	Please indicate to what extent you agree with the following statements: (1 = do not agree at all, 7 = fully agree)				
*Intention_desire*	I would like to supervise and support members with disabilities in the gym.	5.37	1.40	1	7
*Intention_notintend* ^a^	I do not intend to supervise or support members with disabilities in the gym. (reverse coded)	3.11	2.14	1	7
*Intention_plan*	I plan to bring, supervise, and support members with disabilities in the gym.	4.17	1.81	1	7
*Intention_want*	I want to supervise and support members with disabilities in the gym.	5.23	1.48	1	7

exp., experiential; ins., instrumental; des., descriptive; inj., injunctive; aut., autonomy; cap., capacity.

^a^ Items were excluded from further analysis as part of the model adjustment.

The questionnaire began with questions focused on the gym. Respondents provided information about the gym where they currently work or, if not currently employed, the gym where they last worked. This section identified the gym's company structure (*Structure*). The survey also captured the extent to which coaches agreed with statements regarding equipment for people with disabilities (*Equipment*) and accessibility. A mean index was calculated from three items reflecting accessibility (entrance area, training area, and sanitary facilities) to determine overall gym accessibility (*Barrier*).

The next section covered *qualification*(s), with three questions on general qualifications, specific qualifications in group fitness training, and specific qualifications in individual fitness training. Respondents could select from 21 options for the first question, eleven for the second, and 17 for the third, allowing for multiple answers. Responses were classified according to the European Qualification Framework and its adaptation by the German Fitness Instructors Association (44), resulting in a seven-point scale from training (1) to university degree (6), with zero indicating no qualification.

The following block addressed preparation for working with people with disabilities. Coaches indicated whether their fitness training qualification covered supervision and support for members with disabilities (*Topic*) and to what extent other qualifications prepared them for this role (*Preparation*).

Before assessing previous contact with people with disabilities, coaches reported their professional experience as fitness coaches (*Experience*). Two items measured previous contact with people with disabilities: frequency of contact with gym members with disabilities in the gym (*Contact_gym_frequency*), and frequency of contact with people with disabilities outside the gym (*Contact_outside_frequency*). These items provide insights into coaches' experience and frequency of contact both inside and outside the gym.

The items for the theory of planned behavior were constructed based on the corresponding instructions by Fishbein and Ajzen (6). This construction also includes the splitting of the three factors into two sub-factors each. This splitting is justified in terms of content in the theory section.

Attitude was measured using a semantic differential with twelve items, divided into experiential attitude (*Attitude_exp*) and instrumental attitude (*Attitude_ins*). Coaches spontaneously assessed their attitudes toward supervising and supporting members with disabilities. Semantic differentials, used in psychology and social sciences, record attitudes using bipolar adjective pairs (6, 45). They are also used in sporting contexts for various movement tasks (46).

Twelve items captured subjective norms, exploring perceived social pressure from different social groups regarding the supervision and support of gym members with disabilities. Each item assessed the extent to which various groups—such as partners, supervisors, or friends—would support or think the coach should support members with disabilities. These items used a 7-point scale, offering nuanced insights into social influences affecting coaches' intentions and behaviors in fitness environments. By capturing both descriptive norm (*Norm_des*) and injunctive norm (*Norm_inj*), these items help understand the broader social context influencing coaches' actions toward inclusivity.

Items regarding perceived behavioral control (PBC) assess how capable coaches feel and whether they have the resources to support gym members with disabilities. Eight statements evaluate aspects of perceived control, such as time, responsibility, opportunity, and decision-making power (autonomy: *PBC_aut*), as well as knowledge, conviction, skills, and ability (capacity: *PBC_cap*). Coaches indicated their agreement on a 7-point scale, from 1 (do not agree at all) to 7 (fully agree).

Four items measured coaches' intentions regarding supervision and support of gym members with disabilities. Each statement reflected different aspects of intention, such as general interest (*Intention_like*), planning (*Intention_plan*), and desire (*Intention_desire*). The item *Intention_notintend* is reverse-coded, presenting a negative statement to counterbalance positive statements, ensuring data reflect respondents' true intentions and accounting for response biases (47).

The questionnaire concluded with two personal details: *gender* and *age*.

### 3.3 Data analysis

The analysis of data was performed using IBM SPSS 28 and Mplus. Initially, a confirmatory factor analysis (CFA) evaluated the measurement model. Afterwards, a structural equation model (SEM) was developed and tested, as well as a regression analysis.

Given the reliance on self-reported data, common method bias can represent an issue. We conducted Harman's single-factor test by entering all survey items into an exploratory factor analysis to determine whether a single factor accounted for the majority of variance. The results indicated that the first factor accounted for less than 50% of the total variance, suggesting that CMB is unlikely to significantly affect our findings (48).

CFA is an appropriate method because it validates the measurement model based on the TPB framework. Employing CFA establishes construct validity by assessing whether the items effectively measure the three underlying constructs. Additionally, CFA provides various fit indices, such as the Comparative Fit Index (CFI), Root Mean Square Error of Approximation (RMSEA), and Standardized Root Mean Square Residual (SRMR), which help evaluate how well the proposed model aligns with the observed data (49).

To determine which of the three factors influence gym coaches' intentions to supervise individuals with disabilities, SEM is an ideal analytical approach. SEM is particularly suitable because it examines complex relationships between variables within the theory of planned behavior framework. Utilizing SEM analyzes the specific effects of multiple predictor variables on intention. Furthermore, SEM offers the advantage of assessing model fit through various indices, ensuring the proposed model accurately reflects the data and theoretical assumptions. As noted by Kline (50), SEM is particularly effective in social sciences for testing theoretical models involving multiple interrelated constructs.

To explore which contextual factors predict the intention-forming factors determined with SEM, a regression analysis is an appropriate choice. This statistical technique identifies and quantifies relationships between various contextual variables and the intention-forming constructs within the TPB framework. Regression analysis suits this task because it handles multiple predictor variables and assesses their individual contributions to each dependent variable, enabling a detailed understanding of how different contextual factors influence intention formation. By examining coefficients and significance levels, researchers can determine the strength and direction of these relationships. As highlighted by Cohen et al. (51), regression analysis is a powerful tool for investigating causal relationships and testing hypotheses about the influence of contextual variables in social science research. The regression included the predictors which, according to the current state of research, have an influence on the intention or the six underlying factors (see 2.3). In addition to considering the personal control variables (age and gender), the analysis included three external factors—structure, equipment, and barriers—that are managed by the gym rather than the coaches.

## 4 Results

Before presenting the results, the evaluation results of the measurement model are provided.

### 4.1 Measurement model

The model specification process typically involves three steps. Step 1 specifies the measurement model for each respective dimension. Step 2 involves adjustments by excluding items based on insufficient, standardized factor loadings and significances. Items are excluded if their standardized factor loadings fall below 0.70, indicating they only contribute minimally to the respective latent construct (52). Specifically, nine items were excluded in this step. Table 1 highlights these items. In addition to statistical criteria, we considered content validity and theoretical relevance during the exclusion process. This involved thorough discussions within our author team, leveraging our combined expertise to ensure that the retained items adequately represented the constructs of interest. In the final step, individual dimension measurement models merge into an overall measurement model (50).

Results from the confirmatory factor analysis demonstrate a good fit for the constructs of attitude, subjective norm, and perceived behavioral control, with CFIs all above 0.9, indicating these are important factors of intention (53). For detailed fit indices, please refer to Table 2.

**Table 2:** Fit indices for the confirmatory factor analyses.

Model	RMSEA	CFI	SRMR
*Attitude*	0.056	0.966	0.035
*Subjective norm*	0.064	0.963	0.036
*Perceived behavioral control*	0.069	0.977	0.032
*Intention*	0.000	1.000	0.000

### 4.2 Structural equation model

The structural equation model analysis reveals which factors significantly influence intention. The model demonstrates an acceptable fit with an RMSEA of 0.061, a CFI of 0.922, and an SRMR of 0.056. According to Hu and Bentler (54), an RMSEA ≤ 0.06 and an SRMR ≤ 0.08 indicate a good fit, while a CFI ≥ 0.90 is considered acceptable. The standardized regression coefficients indicate that experiential attitude significantly influences intention (*β* = 0.235, *p* = 0.002), along with descriptive norm (*β* = 0.768, *p* < 0.001).

This leads to the confirmation of H1b and H1d.

**Figure 1:**
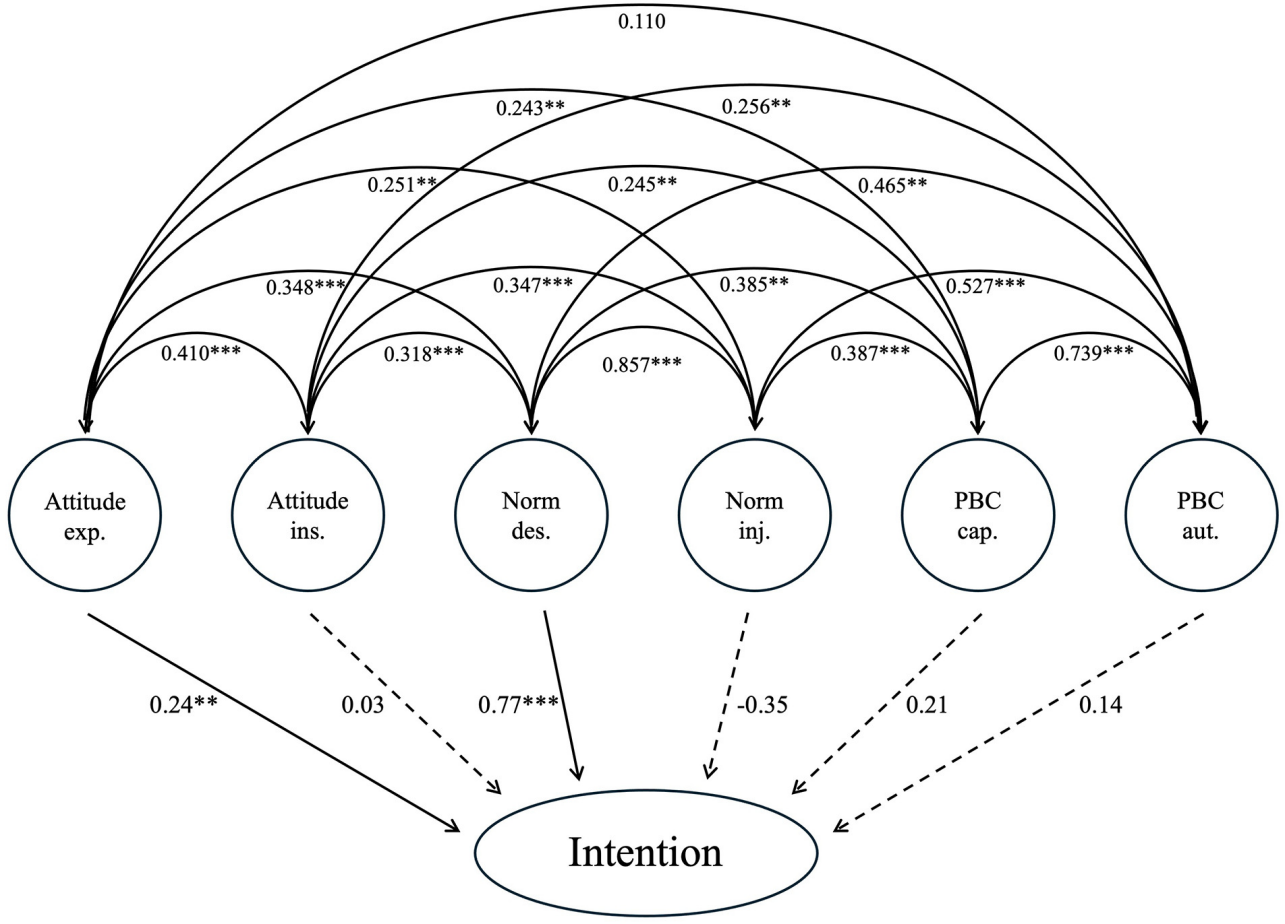
Structural equation model. **p < 0.01; ***p < 0.001.

Other factors did not show significant effects. The model explains 63.3% of the variance in intention. For detailed coefficients, significance levels, and correlations between the six factors, please refer to Figure 1.

### 4.3 Regression analysis

The regression analysis identifies which contextual factors predict the intention-forming factors, specifically experiential attitude and descriptive norm.

The analysis shows that the model predicting experiential attitude explains 13.7% of the variance, with both preparation (*β* = 0.243, *p* < 0.05) and experience (*β* = 0.208, *p* < 0.05) emerging as significant predictors.

For descriptive norm, the model explains 18.9% of the variance, with contact_outside_frequency (*β* = 0.309, *p* < 0.001) being a significant predictor.

This leads to the confirmation of H2c, H2d, and H2f.

Other variables did not show significant effects for either factor. Detailed standardized regression coefficients and explained variances can be found in Table 3.

**Table 3:** Standardized regression coefficients and explained variances of the regression analysis.

Contextual factors	Attitude_exp.	Norm_des.
*Structure_individual* ^REF^	0.051	−0.114
*Structure_micro* ^REF^	0.041	−0.026
*Structure_association* ^REF^	−0.137	−0.061
*Equipment*	−0.053	0.068
*Barrier*	0.024	0.047
*Qualification*	0.097	0.074
*Topic*	−0.181	−0.042
*Preparation*	0.243*	0.149
*Experience*	0.208*	−0.046
*Contact_gym_frequency*	0.013	0.042
*Contact_outside_frequency*	0.197	0.309***
*Gender*	−0.054	0.098
*Age*	−0.096	−0.122
*R^2^*	0.137*	0.189***

REF, reference: structure_chain.

*p < 0.05. ***p < 0.001.

## 5 Discussion

The study aimed to apply the theory of planned behavior by exploring gym coaches' perspectives on including people with disabilities.

Firstly, confirmatory factor analysis confirmed that the TPB constructs—attitude, subjective norm, and perceived behavioral control—significantly influence fitness coaches' intentions. This validation suggests that the TPB framework, as formulated by Fishbein and Ajzen (6), can reliably predict fitness coaches' intentions to work with individuals with disabilities. The confirmed model assumptions justify using the questionnaire in future studies.

Secondly, structural equation model analysis revealed that the descriptive norm and the experiential attitude significantly influence fitness coaches' intentions. This finding highlights the critical role of social influences and experience-based attitudes in shaping intentions. This supports previous studies with (physical education) teachers, where experience (23) and a supportive community (25, 26) impacted intentions and behaviors. These findings suggest that fitness coaches can be compared with physical education teachers regarding the influence of social factors and attitudes on their intentions toward inclusion of people with disabilities.

However, four components of the theory of planned behavior—instrumental attitude, injunctive norm, perceived behavioral control, and subjective norm—did not show a significant association. This non-significance might suggest that, in the context of gyms, the immediate social environment and personal experiences are more influential in shaping intentions than abstract evaluations of outcomes or perceived social pressures. This finding challenges the applicability of TPB components that have been significant in educational and health contexts, potentially highlighting a unique dynamic within fitness environments. Ajzen (7) notes that the relative importance of TPB components can vary depending on the behavioral context, which may explain their limited role here.

Our findings extend previous research by suggesting that in gym settings, descriptive norms and experiential attitudes are more relevant than other TPB components, reflecting a shift in the factors that shape behavioral intentions. Thus, there is a need to adapt TPB models to better align with the specific associations present in fitness environments, where direct social interactions and personal experiences are paramount. Moreover, it is possible that these components interact in complex ways that require further exploration. For instance, Conner and Armitage (55) suggest that perceived behavioral control may be moderated by past behavior or specific situational constraints, such as institutional policies and gym structures, which may limit individual autonomy and relate to intentions. Similarly, Trafimow et al. (56) highlight that while injunctive norms can be strong predictors of intention in some contexts, their association may diminish when descriptive norms take precedence. Additionally, research indicates that subjective norms might be less impactful when individuals prioritize personal experiences over perceived social expectations (57). Instrumental attitude, which involves the evaluation of the outcomes of a behavior, might be less relevant in contexts where emotional and experiential factors are more closely associated with behavioral intentions. According to Fishbein and Ajzen (6), the role of instrumental attitudes can be overshadowed when individuals are more focused on immediate social feedback or personal experiences rather than long-term outcomes.

Lastly, regression analysis suggests that frequent contact with people with disabilities outside the gym, adequate preparation, and experience are crucial predictors of intention-forming constructs. The findings emphasize the importance of engagement beyond the gym environment, suggesting that direct contact with individuals with disabilities in general life plays a significant role in shaping positive intentions toward people with disabilities in the gym context. This finding aligns with the research by Braksiek et al. (58), who found that private contact with individuals with disabilities significantly influenced teachers' attitudes toward inclusive physical education. Moreover, preparation is more effectively achieved through other qualifications rather than formal education. The interviewees stated qualifications such as further training in special education, a degree in social work, and experience in youth welfare. This is in line with Cushion et al. (59), who emphasized the impact of experiential learning and mentorship in sports coaching. They argue that real-world experiences and mentorship offer context-specific and adaptable training that is often more effective than traditional educational programs. Additionally, the longer one works as a coach, the more likely a positive experiential attitude develops. This finding is consistent with research in educational settings, where Oh et al. (24) found that teachers with more years of experience often develop more positive attitudes toward inclusive education due to increased exposure and familiarity with diverse student needs. Similarly, Tiwari et al. (22) highlighted that experience in teaching enhances confidence and positive attitudes toward inclusive practices. These parallels suggest that accumulated experience in coaching can similarly lead to more positive attitudes toward integrating gym members with disabilities. However, the relatively low explained variance in the regression analysis suggests that additional factors may be associated with the intentions of fitness coaches that were not captured in this study. This is a common challenge in research involving the TPB, where the explanatory power of models is often “disappointingly low”, as noted by Heuer and Kolvereid (60), in their study on entrepreneurship education. Additionally, the reliance on cross-sectional data may contribute to unexplained variance, as it can introduce bias by capturing both independent and dependent variables from the same source.

The study's findings have significant implications for policy and practice in inclusive fitness coaching. Comprehensive training programs that emphasize practical and experiential learning should be integrated into certification processes to equip coaches with the skills needed to support individuals with disabilities. Specifically, targeted training should include components such as disability etiquette, adaptive exercise techniques, and strategies for creating inclusive environments. Disability etiquette training can help coaches to communicate effectively and respectfully with individuals with disabilities, reducing barriers of participation (39). Adaptive exercise techniques are essential for modifying workouts to meet the diverse needs of gym members, ensuring that individuals with different abilities can participate fully (61). Additionally, coach education should emphasize the importance of creating an inclusive gym culture that values diversity and promotes engagement, as this has been shown to enhance participation and satisfaction among all members (33). This idea aligns with Sharon-David et al. (4), who highlight the need to address physical accessibility and create welcoming environments in gyms to overcome participation barriers. Fitzgerald et al. (33) found that individuals with disabilities often report feelings of marginalization in gym environments, which can lead to decreased motivation to engage in physical activities. To address this issue, it is crucial to explore how coaches can be equipped to understand and support different types of motivation among gym participants. The classification system of motivational behaviors proposed by Ahmadi et al. (62) offers a valuable framework for coaches to enhance their ability to motivate and engage individuals with diverse needs. By adopting strategies from this framework, coaches can create fitness environments that foster greater participant engagement and reduce feelings of marginalization, thereby promoting a more inclusive culture. This aligns with the study's broader recommendations for comprehensive coach education programs and policy changes, further enhancing the practical application of inclusive practices in gyms.

Engagement with people with disabilities is crucial, and organizations should facilitate community outreach and partnerships with disability advocacy groups, as suggested by Richardson et al. (35–37), who emphasize the importance of social support in increasing gym participation. Such initiatives foster empathy and understanding, which are essential for effective inclusion. At the organizational level, the perceptions noted by Lesch et al. (38) indicate a need for a shift in management attitudes toward disability inclusion. Policy changes and incentives could encourage gyms to adopt inclusive practices, supported by training that incorporates elements of autonomy, belongingness, and engagement, as advocated by Martin Ginis et al. (40). Despite positive intentions, a gap remains in implementing inclusive practices effectively. Organizations should provide support and resources, including adaptive equipment, to enhance coaches' capacity to accommodate diverse needs, consistent with Rimmer et al. (39), who stress the importance of equipping staff with the necessary tools to support individuals with disabilities. Integrating the principles of Universal Design for Learning (UDL) throughout these training and policy recommendations can further enhance their effectiveness. UDL's focus on providing multiple means of engagement, representation, and action and expression (63) aligns well with the adaptive and inclusive strategies outlined in this study. By embedding UDL principles, fitness environments can become more accessible and responsive to the diverse needs of all members, thereby promoting an inclusive culture that empowers everyone to participate fully. These efforts can lead to broader societal benefits, such as increased social integration and improved mental health outcomes, as discussed by Fitzgerald et al. (33).

While the study provides valuable insights into fitness coaches' intentions regarding the inclusion of individuals with disabilities, some methodological limitations should be noted. Firstly, reliance on self-reported data may introduce bias, as coaches might respond in socially desirable ways rather than reflecting their true intentions (64). To address this concern, we already conducted Harman's single-factor test to assess the presence of common method bias. Secondly, the use of the same dataset for both model validation and hypothesis testing is a limitation. This approach may inflate the validity of the results, as it does not account for potential overfitting or sample-specific biases (65). Future studies should consider using separate datasets or different participant groups to validate models and test hypotheses, which would enhance the robustness and generalizability of the results. Moreover, the limited sample size in the current study, may also impact the extent to which the findings can be generalized (66). The study also focused on a specific context, where intentions may differ in other contexts and be influenced by the country's overall attitude and implementation of inclusion (i.e., Germany). As highlighted by Tah et al. (67), national policies on inclusive education often lack a clear-cut definition and vary in stakeholder involvement, reflecting differences in how inclusion is constructed across various national contexts. To address these limitations, future research could employ longitudinal designs to track changes in coaches’ behavioral intentions over time, what would provide insights into how intentions develop and persist (68). Additionally, cross-national comparisons could be conducted to examine how cultural and policy differences influence fitness coaches' intentions toward inclusion (69). Furthermore, incorporating mixed methods approaches could provide a more comprehensive understanding by combining quantitative data on intentions with qualitative insights into how these intentions translate into actual inclusive practices (70). These approaches would enhance the understanding and practical application of inclusive practices in gyms across diverse settings.

In summary, while fitness coaches express strong intentions to support individuals with disabilities, targeted training and development programs are needed to bridge the gap between intention and actual practice. The study underscores the importance of external engagement and comprehensive training initiatives to foster a more supportive and inclusive fitness culture.

## Data Availability

The data supporting the findings of this study are available from the corresponding author upon request. However, due to confidentiality agreements with survey respondents, the data cannot be made publicly available or shared without restrictions.
