# Supplementary material for: Predicting fitness coaches’ intentions to include persons with disabilities in gyms: an application of the theory of planned behavior
Source: Front Sports Act Living. 2025 Jul 24;7:1608703. doi: 10.3389/fspor.2025.1608703 (PMC12329752; doi:10.3389/fspor.2025.1608703)
Supplement: Supplementary file 1 [file Datasheet1.pdf]

UNIVERSITÄT BIELEFELD
Faculty of Psychology and Sports Science

1. Information

Dear Sir or Madam,

Thank you for your interest in this survey.

Study Purpose

This survey is part of a research project supported by the State Chancellery of North Rhine-Westphalia on the inclusion of people with disabilities in gyms, carried out by Prof. Dr. Pamela Wicker and Prof. Dr. Bernd Gröben (Bielefeld University).

Procedure and Content of the Survey

The survey takes about 10 minutes and is aimed at coaches and instructors who currently work in a gym or have worked in one previously. The minimum age for participation in the survey is 16 years.

In the survey, we are interested in your views on the supervision and support of members with disabilities in the gym. In addition, information about the gym, your qualifications, your previous experience in contact with people with disabilities, as well as your age, gender, and income will be collected.

We define people with disabilities as those who have physical, mental, intellectual, or sensory impairments that are very likely to prevent them from participating in society on an equal basis with others for longer than six months.

Data Protection, Data Access, and Processing Duration

The data will only be collected anonymously. It will therefore not be possible to identify you, or other individuals based on your answers or data. The anonymized data will be stored on a university-internal, password-protected server and kept for 10 years in accordance with Bielefeld University's retention policy, allowing for future research use after the project's conclusion.

Data Publication

Your data will be used exclusively for scientific purposes and will be treated in strict confidence. The presentation of the results to third parties (e.g., in scientific publications or

lectures) does not allow any conclusions to be drawn about individual individuals due to the anonymous collection of your answers.

Risks

No negative consequences or special burdens are to be expected as a result of participation.

Voluntary Participation

Participation in the survey is voluntary. You may cancel the survey at any time without giving reasons and without incurring any disadvantages.

Raffle of Vouchers

At the end of the survey, you can take part in a prize draw for four Sport Scheck vouchers worth €50 each. You can take part in the prize draw if you have completed the survey in full. Registration for the prize draw is voluntary and requires you to provide your email address and consent to participate at the end of the survey. The email address will be stored separately from the rest of your answers so that it is not possible to link your answers to your email address.

Contact Information

If you have any questions, please feel free to contact Prof. Dr. Bernd Gröben (bernd.groeben@uni-bielefeld.de) or Dr. Christopher Meier (christopher.meier@uni-bielefeld.de).

2. Declaration of consent for participation

I have read and understood the information for participating in the survey. I feel sufficiently informed and have had the opportunity to ask questions.

I am aware that my participation in the survey is voluntary and that I will not face any disadvantages if I refuse to give my consent. I can withdraw my consent at any time by canceling the survey. No justification is required for this withdrawal and there will be no disadvantages whatsoever. Once the completed questionnaire has been submitted, participation can no longer withdrawn, as the anonymized data can no longer be assigned to individual participants.

- ☐ Yes, I agree and would like to take part in the survey.
- ☐ No, I do not agree and would like to end the survey.

3. Details of the gym

All of the following information refers to the gym where you currently work. If you work in several gyms, please refer to the gym where you work the most hours per week. If you are not currently working at a gym, please refer to the gym where you last worked.

Which organizational structure can the gym be assigned to?

- ☐ Chain operation (fitness companies with five or more facilities)
- ☐ Individual operation (fitness companies with one to four facilities)
- ☐ Micro studio (fitness companies with less than $200m^2$ and deliberately limited offer for specific target groups)
- ☐ Gym of an association

Please provide the approximate number of members of the gym.

____

Please provide what the gym offers. (multiple answers possible)

- ☐ Training area operation
- ☐ General fitness courses (e.g. Body Pump, Pilates, Spinning)
- ☐ Health-orientated courses (e.g. Rehabilitation Sports, Back Fitness)
- ☐ Specialization of the gym (e.g. Kieser, EMS, CrossFit)
- ☐ Special training equipment for people with disabilities
- ☐ Special offers for people with disabilities
- ☐ Other offers

What does the gym specialize in?

____

What special training equipment is available in the gym for people with disabilities?

____

What special offers are there for people with disabilities in the gym?

____

What other offers are there in the gym?

____

Please indicate to what extent you agree with the following statements:

	1 do not agree at all	2	3	4	5	6	7 fully agree
The entrance area of the gym is barrier-free.	□	□	□	□	□	□	□
The training area of the gym is barrier-free.	□	□	□	□	□	□	□
The sanitary facilities of the gym are barrier-free.	□	□	□	□	□	□	□
The gym has specific equipment (e.g. training equipment) for people with disabilities.	□	□	□	□	□	□	□

4. Information on qualifications

What qualification(s) do you have? (multiple answers possible)

- ☐ Athletics coach A license
- ☐ Training as a sports and fitness manager
- ☐ Fitness specialist
- ☐ Specialist consultant for fitness
- ☐ Specialist in prevention and health promotion
- ☐ Fitness coach C license
- ☐ Fitness coach B license
- ☐ Fitness coach A license
- ☐ Instructor for group training
- ☐ Teacher for prevention and health promotion
- ☐ Teacher for wellness and health
- ☐ Fitness teacher
- ☐ Teacher for preventive and rehabilitative training
- ☐ Physiotherapist
- ☐ Degree in sports science
- ☐ Degree in the fitness sector (e.g. BA Fitness Training, MA Prevention and Health Management)
- ☐ Coach A license for strength and functional training
- ☐ Instructor license (DOSB)
- ☐ Coach license (DOSB)
- ☐ Further qualification(s)
- ☐ None

Which DOSB instructor license do you have?

___

Which DOSB coach license do you have?

___

What other qualification(s) do you have?

___

What specific qualification(s) in the field of fitness group training do you have? (multiple answers possible)?

- ☐ Athletic coach
- ☐ Athletics coach for competitive sports
- ☐ EMS coach
- ☐ Health coach
- ☐ Kieser training instructor
- ☐ Competitive sports body coach
- ☐ Milon coach
- ☐ Coach for cardio fitness
- ☐ Coach for fitness training in cancer aftercare
- ☐ Coach for free weight and kettlebell training
- ☐ Coach for bodyweight and sling training
- ☐ Coach for preventive back training
- ☐ Coach for rehabilitative strength training
- ☐ Coach for sports rehabilitation
- ☐ Coach for equipment-based strength training
- ☐ Further qualification(s)
- ☐ None

What other qualification(s) in the field of individual fitness training do you have?

___

What specific qualification(s) do you have for the supervision and support of people with disabilities?

- ☐ Instructor C - popular sport - disabled sport (DBS)
- ☐ Instructor B - popular sport - disabled sport (DBS)
- ☐ Instructor B - prevention sport - for people with disabilities (DBS)
- ☐ Instructor B - rehabilitation sports - orthopedics (DBS)
- ☐ Instructor B - rehabilitation sports - internal medicine (DBS)
- ☐ Instructor B - rehabilitation sports - sensory (DBS)
- ☐ Instructor B - rehabilitation sports - neurology (DBS)
- ☐ Instructor B - rehabilitation sport - mental disability (DBS)
- ☐ Instructor B - rehabilitation sports - psychiatry (DBS)

☐ Coach C - competitive sport - disabled sport (DBS)
☐ Coach B - competitive sport - disabled sport (DBS)
☐ Coach A - competitive sport - disabled sport (DBS)
☐ Further qualification(s) for the supervision and support of people with disabilities
☐ None

What other qualification(s) do you have for the supervision and support of people with disabilities?

____

5. Thematization of supervision and support for members with disabilities during vocational qualification

Was the topic of supervision and support for members with disabilities covered during your fitness training qualification(s)?

☐ Yes
☐ No

My fitness training qualifications have prepared me for the supervision and support of members with disabilities in gyms.

1<br>do not agree<br>at all	2	3	4	5	6	7<br>fully agree
☐	☐	☐	☐	☐	☐	☐

Was the topic of supervision and support of people with disabilities covered during your further qualification(s)?

☐ Yes
☐ No

My other qualifications have prepared me for the supervision and support of members with disabilities in gyms.

1<br>do not agree<br>at all	2	3	4	5	6	7<br>fully agree
☐	☐	☐	☐	☐	☐	☐

Have you attended any training courses, seminars, etc. on the topic of supervision and support for members with disabilities in gyms?

- ☐ Yes
- ☐ No

How many training courses have you attended on the topic of supervision and support for members with disabilities in gyms?

____

I would like to attend training courses on the supervision and support of members with disabilities in gyms.

1<br>do not agree at all	2	3	4	5	6	7<br>fully agree
☐	☐	☐	☐	☐	☐	☐

6. Information on professional experience

Do you currently work in a gym?

- ☐ Yes
- ☐ No

How many hours per week do you work at this gym?

____

How many years have you worked in gyms?

____

7. Previous experience in contact with people with disabilities

Have you ever had contact with members with disabilities at the gym?

- ☐ Yes
- ☐ No

How intensive was the contact with members with disabilities in the gym?

1 superficial	2	3	4	5	6	7 narrow
☐	☐	☐	☐	☐	☐	☐

How often do you come in contact to members with disabilities in the gym?

- ☐ Daily
- ☐ Several times per week
- ☐ Once a week
- ☐ Several times a month
- ☐ Once a month
- ☐ Several times a year
- ☐ Once a year
- ☐ Less than once a year

Have you ever had contact with people with disabilities outside the gym?

- ☐ Yes
- ☐ No

Where did this contact take place outside the gym? (multiple answers possible)

- ☐ Work outside the gym
- ☐ Professional qualification
- ☐ Private
- ☐ School time
- ☐ Other contact

How intense was your contact with people with disabilities outside the gym?

1 superficial	2	3	4	5	6	7 narrow
☐	☐	☐	☐	☐	☐	☐

How often do you come into contact with people with disabilities outside the gym?

- ☐ Daily
- ☐ Several times per week
- ☐ Once a week
- ☐ Several times a month
- ☐ Once a month
- ☐ Several times a year
- ☐ Once a year

☐ Less than once a year

8. Assessments of the supervision and support of members with disabilities in the gym

Please do not think too long about the following assessments, we are interested in your spontaneous judgement on the following statement:

Supervising and supporting members with disabilities in the gym is...

good	◻	◻	◻	◻	◻	◻	◻	bad
boring	◻	◻	◻	◻	◻	◻	◻	exciting
wrong	◻	◻	◻	◻	◻	◻	◻	right
repulsive	◻	◻	◻	◻	◻	◻	◻	attractive
superfluous	◻	◻	◻	◻	◻	◻	◻	necessary
satisfactory	◻	◻	◻	◻	◻	◻	◻	unsatisfactory
unimportant	◻	◻	◻	◻	◻	◻	◻	important
pleasant	◻	◻	◻	◻	◻	◻	◻	unpleasant
appealing	◻	◻	◻	◻	◻	◻	◻	disgusting
sensible	◻	◻	◻	◻	◻	◻	◻	pointless
unfavorable	◻	◻	◻	◻	◻	◻	◻	favorable
harmless	◻	◻	◻	◻	◻	◻	◻	frightening

Please indicate to what extent you agree with the following statements:

	1 do not agree at all	2	3	4	5	6	7 fully agree
My colleagues think I should supervise and support members with disabilities in the gym.	◻	◻	◻	◻	◻	◻	◻
My partner would supervise and support members with disabilities at the gym.	◻	◻	◻	◻	◻	◻	◻
My supervisor would recognize, supervise, and support members with disabilities in the gym.	◻	◻	◻	◻	◻	◻	◻
My friends think I should supervise and support members with disabilities at the gym.	◻	◻	◻	◻	◻	◻	◻
Members of the gym without disabilities think I should supervise and support members with disabilities in the gym.	◻	◻	◻	◻	◻	◻	◻
My family members would supervise and support members with disabilities at the gym.	◻	◻	◻	◻	◻	◻	◻
My partner thinks I should have members, support, and supervise people with disabilities in the gym.	◻	◻	◻	◻	◻	◻	◻
My colleagues would supervise and support members with disabilities in the gym.	◻	◻	◻	◻	◻	◻	◻
Members of the gym without disabilities would supervise and support members with disabilities in the gym.	◻	◻	◻	◻	◻	◻	◻
My supervisor thinks I should supervise and support members with disabilities in the gym.	◻	◻	◻	◻	◻	◻	◻

My friends would include, supervise, and support members with disabilities in the gym.	☐	☐	☐	☐	☐	☐	☐
My family members think I should supervise and support members with disabilities at the gym.	☐	☐	☐	☐	☐	☐	☐

Please indicate to what extent you agree with the following statements:

	1 do not agree at all	2	3	4	5	6	7 fully agree
I know how to supervise and support members with disabilities in the gym.	☐	☐	☐	☐	☐	☐	☐
I have the time to supervise and support members with disabilities in the gym.	☐	☐	☐	☐	☐	☐	☐
I am convinced that I can supervise and support members with disabilities in the gym.	☐	☐	☐	☐	☐	☐	☐
I am responsible for supervising and supporting members with disabilities in the gym.	☐	☐	☐	☐	☐	☐	☐
I have the opportunity to include, supervise, and support members with disabilities in the gym.	☐	☐	☐	☐	☐	☐	☐
I have the skills to supervise and support members with disabilities in the gym.	☐	☐	☐	☐	☐	☐	☐
It is my decision to supervise and support members with disabilities in the gym.	☐	☐	☐	☐	☐	☐	☐
I am able to supervise and support members with disabilities in the gym.	☐	☐	☐	☐	☐	☐	☐

Please indicate to what extent you agree with the following statements:

	1 do not agree at all	2	3	4	5	6	7 fully agree
I would like to supervise and support members with disabilities in the gym.	☐	☐	☐	☐	☐	☐	☐
I do **not** intend to supervise or support members with disabilities in the gym.	☐	☐	☐	☐	☐	☐	☐
I plan to bring, supervise, and support members with disabilities in the gym.	☐	☐	☐	☐	☐	☐	☐
I want to supervise and support members with disabilities in the gym.	☐	☐	☐	☐	☐	☐	☐

9. Personal details

What gender do you identify with?

- ☐ Woman
- ☐ Man
- ☐ Diverse

How old are you?

____

What type of town or municipality do you currently live in?

- ☐ Village (under 5000 inhabitants)
- ☐ Small town (5000-19999 habitants)
- ☐ Medium-sized City (20000-99999 inhabitants)
- ☐ Large City (from 100000 inhabitants)

If you are not currently working at a gym, please refer to the gym where you last worked for the following questions.

Why do you work in the gym? (multiple answers possible)

- ☐ Contact(s) to the gym
- ☐ Proximity to place of residence
- ☐ Financial reasons
- ☐ Professional development opportunities
- ☐ Match my qualifications
- ☐ Other reasons

What other reasons do you have for working at the gym?

____

What is your personal monthly net income from your gym employment?

- ☐ Up to 250€
- ☐ 251-500€
- ☐ 501-1000€
- ☐ 1001-1500€
- ☐ 1501-2000€
- ☐ 2001-2500€
- ☐ 2501-3000€
- ☐ 3001-3500€
- ☐ 3501-4000€
- ☐ More than 4000€

What is your personal monthly total net income?

- ☐ Up to 500€
- ☐ 501-1000€
- ☐ 1001-1500€
- ☐ 1501-2000€
- ☐ 2001-2500€
- ☐ 2501-3000€
- ☐ 3001-3500€
- ☐ 3501-4000€
- ☐ More than 4000€

10. Thank you four your participation!

Thank you for taking the time to participate in this survey.

Please click on Send and then confirm with [Ok] to complete the survey.

You will then have the opportunity to take part in a prize draw for four Sport Scheck vouchers worth €50 each. Registration for the prize draw is voluntary and requires you to provide your email address and consent to participate.

If you have any questions or suggestions regarding this survey, please contact Prof. Dr. Bernd Gröben (bernd.groeben@uni- bielefeld.de) or Dr. Christopher Meier (christopher.meier@uni-bielefeld.de).
